# Elastic Adhesive Plaster

**Published:** 1878-10

**Authors:** 


Elastic Adhesive Plaster.—For some time I have been trying to find an elastic covering that, being attached to the skin, would move to the movements of that membrane and the parts beneath it without causing an unbearable sensation of stiffness or an uncomfortable wrinkling. As there was nothing in our market to suit me I prucured some india rubber, and giving it a coat of plaster, such as recommended in Griffith's Formulary under the name of Boynton's adhesive plaster (lead plaster one pound, rosin six drachms,) I found the material I wished. After using it as a simple covering for cases of psoriasis, intertrigo, etc., I extended its use to incised wounds, abscesses, etc., and found it invaluable. Placing one end of a strip of the plaster upon one lip of the end of the wound and then streching the rubber and fastening the other end to the opposite lip of the wound I had perfect apposition of the several parts, the elastic rubber acting continually to draw and keep the parts together. When I have been unable to get the sheets of rubber I have used the broad letter bands (sold by all stationers) by giving them a coat of plaster. I do not know if this kind of plaster has ever been placed before the profession. If it has I can do no harm in again calling the attention of surgeons to it. If it has not, I feel assured that any one who uses it once will never again be without it, as it is cleanly, certain and satisfactory in its action, and fulfils indications that no other plaster does or can.—*Boston Med. and Surg. Jour.*

